# Systemic Arthritis in Children: A Review of Clinical Presentation and Treatment

**DOI:** 10.1155/2012/271569

**Published:** 2011-12-25

**Authors:** R. Gurion, T. J. A. Lehman, L. N. Moorthy

**Affiliations:** ^1^Division of Pediatric Rheumatology, Rainbow Babies & Children's Hospital, University Hospitals Case Medical Center, 11100 Euclid Avenue, Cleveland, OH 44106, USA; ^2^Division of Pediatric Rheumatology, Hospital for Special Surgery and Clinical Pediatrics Weill Medical Center, Cornell University, 535 E 70 St, New York, NY 10021, USA; ^3^Division of Pediatric Rheumatology, Department of Pediatrics, University of Medicine and Dentistry of NJ-Robert Wood Johnson Medical School, 89 French Street, New Brunswick, NJ 08901, USA

## Abstract

Systemic juvenile idiopathic arthritis (sJIA) constitutes a small part of juvenile idiopathic arthritis (JIA), yet has a disproportionally higher rate of mortality. Despite being grouped under JIA, it is considered to be a multifactorial autoinflammatory disease. The objective of this paper is to review the epidemiology, pathogenesis, genetics, clinical manifestations, complications, therapy, prognosis, and outcome of sJIA. The presentation and clinical manifestations of sJIA have not changed much in the past several decades, but the collective understanding of the pathogenesis and the development of new targeted therapies (particularly the biologic agents) have transformed and improved the disease outcome for children with sJIA.

## 1. Introduction

In 1897, Sir George Fredrick Still described 22 children, 12 of whom had a unique constellation of symptoms that included chronic arthritis, adenopathy, splenomegaly, and fevers [1]. Initially bearing his name, and later known by other names (systemic juvenile rheumatoid arthritis, systemic juvenile chronic arthritis), this entity is now known as systemic arthritis [2]. To allow for improved identification and research the International League of Associations of Rheumatology (ILAR) proposed a classification for JIA [2, 3]. To fulfill the criteria for systemic juvenile idiopathic arthritis (sJIA) a child must be under 16 years of age and have “arthritis in one or more joints with or preceded by fever of at least 2 weeks' duration that is documented to be daily (“quotidian”) for at least 3 days and accompanied by one or more of the following: (1) evanescent (nonfixed) erythematous rash, (2) generalized lymph node enlargement, (3) hepatomegaly and/or splenomegaly, (4) serositis” [3]. Exclusions include “(a) psoriasis or a history of psoriasis in the patient or a first-degree relative, (b) arthritis in an HLA-B27 positive male beginning after the 6th birthday, (c) ankylosing spondylitis, enthesitis-related arthritis, sacroiliitis with inflammatory bowel disease, Reiter's syndrome, or acute anterior uveitis, or a history of one of these disorders in a first-degree relative, (d) the presence of IgM rheumatoid factor on at least 2 occasions at least 3 months apart” [3]. Despite being included under the inclusive umbrella of juvenile idiopathic arthritis (JIA), it is likely that sJIA is a different disease, for it appears to be unlike the other forms of JIA both in clinical presentation and its pathogenesis [4] (refer to section under pathogenesis). In the following sections we will review the epidemiology, pathogenesis, genetics, clinical manifestations, complications, therapy, prognosis, and outcome of sJIA.

## 2. Age of Onset, Gender and Ethnicity

By definition, sJIA can present at any point until the age of 16; however, in a recent study by Behrens et al., 74 out of 136 patients presented between 0–5 years of age, and age 2 was the most common age at presentation (*n* = 17) [5]. Several studies showed that gender distribution is roughly equal [5, 6]. Ethnic composition seen in sJIA patients from Behrens et al.'s study parallels that of the population in the state of Pennsylvania (with 82% Caucasians and 14% African Americans) [5].

## 3. Incidence and Prevalence

In a recent study by Modesto et al., the prevalence of sJIA was 3.5 per 100,000 [7]. When reviewing older literature, 10–20% of the cases of juvenile rheumatoid arthritis (JRA) was comprised of systemic disease [8]; we are awaiting data from more recent studies using the current classification system. Disproportionately, sJIA contributes about two-thirds of the total mortality rate in JIA [9]. The incidence of sJIA ranges between 0.4–0.9 per 100,000 per year (Table 1) [7, 10–15].

## 4. Pathogenesis and Genetics

Cytokine dysregulation is seen in sJIA. While interferon *γ* levels are decreased, proinflammatory cytokines such as tumor necrosis factor-*α* (TNF-*α*), interleukin-6 (IL-6), interleukin-8, monocyte chemoattractant protein-1, E-selectin, and intracellular adhesion molecules levels are elevated in sJIA [16–21]. Recently, the role of interleukin-1*β* (IL-1*β*) in sJIA received attention. Excess IL-1*β* can result in fever, anorexia, pain hypersensitivity, joint destruction, vasculitis, and thrombosis [22]; its dysregulation can lead to the clinical and laboratory findings of sJIA. In Pascual et al.'s study, culturing healthy peripheral blood mononuclear cells with serum of sJIA patients caused an increase in IL-1 secretion; an increased production of IL-1*β* protein from mononuclear cells of active sJIA patients was also seen [23]. IL-1*β* appears to have a pivotal role and may be responsible for the elevation in IL-6 [23]. 

IL-6 has an important role in affecting the systemic manifestations as well as arthritis in sJIA. Elevation of IL-6 in both peripheral blood and synovial fluid is seen; its expression seems to correlate with disease activity and parallel the fever curve [24]. Acute phase reactants (such as C-reactive protein (CRP), serum amyloid A, fibrinogen, and ferritin) are stimulated by IL-6 [25]. It appears to be responsible for the anemia seen in sJIA, as well as promote the production of hepcidin [26]. Hepcidin is produced by the liver and is responsible for transmembrane iron transport; when elevated, it prevents the release of iron from the macrophages, hepatocytes, and enterocytes to the plasma, thus causing a decrease in serum iron levels [26]. In addition, IL-6 may activate osteoclasts and cause osteoporosis, as well as instigate cartilage damage [27].

Other cytokines that may play a role in sJIA are interleukin-18 (IL-18) [28], myeloid-related protein (MRP)-8 and MRP-14 [29, 30], macrophage migratory inhibitory factor (MIF) [31], and interleukin 4-1098 T/G polymorphism [32]. In addition, dysregulation in the expression of anti inflammatory cytokine interleukin-10 (IL-10) (via promoter polymorphism) seems to play an important part in sJIA [33]. 

Innate immune abnormalities in sJIA make it likely to be grouped with the autoinflammatory diseases [34], and indeed, according to the fourth international congress on the systemic autoinflammatory diseases, sJIA is a complex multifactorial autoinflammatory disease [35]. The lack of strong major histocompatibility complex association can be seen both in sJIA and autoinflammatory diseases [34]. 

Pyrin (also known as marenostrin) is a 781 amino acid protein encoded by the familial Mediterranean fever gene (MEFV) found on chromosome 16p [36, 37]. Pyrin plays a role in the downregulation of inflammation [38, 39]. A registry of MEFV gene mutations is kept in the online database Infevers [40], and approximately 180 sequence alterations have been identified, out of which, 5 mutations are the most common ones [41]. When MEFV gene mutations occur, pyrin's function is compromised, and uncontrolled inflammation is potentiated [39]. It is not surprising that some of the genetic defects seen in sJIA are also seen in the autoinflammatory syndromes [39, 42], and particularly in FMF, where mutations in the MEFV gene are present [43]. In ethnic groups where FMF is common, the following diseases have an increased rate of MEFV gene mutations (compared with an ethnically matched population): Polyarteritis nodosa [44], Henoch-Schönlein purpura [45], and Behçet's disease [46–49]. A higher rate of MEFV gene mutations was seen in patients with sJIA in comparison with ethnically matched population (*P* < 0.01) [39]. Interestingly, even when only one allele is affected by mutations or polymorphism, subclinical inflammation can be seen [50, 51]. It is possible that mutations in the MEFV gene prompt a carrier to either develop sJIA or have a more severe course. In a recent study by Ayaz, the MEFV mutation frequency in sJIA patients was seen in 14.28% (significantly higher than in the general population (*P* < 0.01)); the most common mutation was M694, which appeared in frequency of 10% [39].

It has been postulated that a genetic association between sJIA and macrophage activation syndrome (MAS) exists via a mutated perforin gene (PRF1) [52–54] and polymorphism of both MUNC13-4 [55] and interferon regulatory factor 5 (IRF5) [56] genes. However, Donn et al. studied genes known to be associated with the familial form of hemophagocytic lymphohistiocytosis (HLH) (HLH and MAS will both be discussed more under the section on complications) and did not see an increased susceptibility to sJIA [57]. Only a limited number of genes were analyzed, and furthermore, the genetic association of MAS in an existing sJIA patient was not studied [57].

## 5. Features at Presentation

The most common presenting feature is fever, followed by arthritis and rash. Less frequent are lymphadenopathy, pericarditis, and hepatosplenomegaly [5, 58]. Most patients present with laboratory findings indicative of inflammation: elevated erythrocyte sedimentation rate (ESR) and CRP [5, 58], leuko- and thrombocytosis, and elevation in liver transaminases, as well as anemia [5]. Elevation of D-dimers [5, 59], ferritin, and aldolase [5] is seen.

Some patients' initial presentation of sJIA is that of life-threatening MAS (discussed further under complications). The clinical findings are very similar to those of both sJIA and sepsis, with unremitting fever ≥ 38°C, central nervous system manifestations, hemorrhage, lymphadenopathy, hepatosplenomegaly, rash, serositis, and myocarditis [60]. The laboratory findings include thrombocytopenia, hyperferritinemia, elevated liver enzymes, leukocytosis, normal or decreased ESR, hypofibrinogenemia, and hypertriglyceridemia [61].

## 6. Clinical Manifestations

### 6.1. Fever

Fever is the most common symptom at time of initial presentation. According to Behrens et al., 98% of patients present with fever [5], and in a cohort study from the United Kingdom, France, and Spain, 100% of patients presented with fever (this was an inclusion criteria) [58]. Classically, it has been described as a quotidian fever that spikes to greater than 39°C once or twice daily, typically occurring in the evening [8, 62–64]. Although the quotidian pattern is one of the ILAR criteria for diagnosis of sJIA, Behrens et al.'s study showed different patterns. The classic pattern is only seen in 37% of the patients during initial presentation; others exhibit morning fevers (12%), bi-daily fevers (15%), intermittent fevers (27%), and unremitting fevers (5%) [5], as well as not reaching 39°C [5, 62]. In addition to the fevers, some children rapidly defervesce and attain subnormal temperatures [8, 64, 65]. While the child is febrile, other symptoms such as arthritis, rash or serositis can worsen and cause significant disturbance of daily life; however, once the child has defervesced, it is not unusual to see a resumption of regular activities [8, 62, 66].

### 6.2. Musculoskeletal

Arthritis is the second most common presenting symptom [5], and arthralgias can precede the arthritis [65, 66]. According to Behrens et al., 88% of children presented with arthritis [5]. In those cases where arthritis was not found initially, it typically appeared within a few months; infrequently the arthritis will not present until several years later [8]. In Behrens et al.'s study, equal distribution between polyarticular and oligoarticular patterns was noted at presentation (41% polyarticular, 40% oligoarticular, and 7% monoarticular presentation) [5]; however, in a European study the ratio differs, and an oligoarticular pattern is found on presentation twice as often as a polyarticular pattern [58]. The wrists, knees, and ankles are primarily the most commonly involved joints on initial presentation [5, 8]. Despite being mostly asymptomatic, temporomandibular joint arthritis can also be seen [67, 68]. Over the course of the disease, chronic progressive arthritis is seen in approximately one third to one half of the patients [65, 69], and ultimately polyarticular joint involvement is found in most of these cases [8]. Cervical spine arthritis as well as hip arthritis (often bilateral and destructive) can also be seen [8]. 

Another musculoskeletal manifestation of sJIA is the development of synovial cysts [70–73]. These cysts occur more frequently in the upper extremities [70]; they normally resolve on their own, but they may rupture and present as pseudothrombophlebitis [8]. Lymphedema can also occur as a rare musculoskeletal manifestation [8, 74]. It is likely that inflammation of the lymphatic vessels causes this painless swelling [8]. For the most part, pharmacological treatment is not indicated, but compression stocking may improve the lymphedema [8]. 

### 6.3. Rash

Sir George Fredrick Still himself did not describe a rash in the depiction of the disease. It was not until more than 50 years later that attention was given to this unique finding [66, 75]. Evanescent in nature and bright salmon pink in color, this rash is morbilliform, macular, often with central clearing, and tends to be migratory and widespread [64, 75, 76]. It initially emerges mostly on the limbs and trunk and less on the face, neck, palms, and soles [66, 75, 76]. The rash is fleeting (vanishes within a few minutes to a few hours), and it correlates with the acute febrile episodes [64, 75, 76]. According to Behrens et al. 81% of patients presented with a rash [5]. Modesto et al., reported that a rash was seen in 89% of patients with good prognosis and 79% in patients with bad prognosis [58]. Most often the rash is nonpruritic [76], but in about 5% of the patients pruritus does occur [77, 78]. In addition, the Koebner phenomenon (emergence of linear distribution of lesions next to site of injury) can occur [75, 76]. 

During the height of the rash, histological findings reveal only sparse perivascular infiltration of mononuclear cells and neutrophils [75, 76]; normal skin biopsy is seen in patients whose rash had resolved [76]. Just as in other inflammatory processes (such as psoriasis, lichen planus, cutaneous lupus erythematosus, or wound healing [79, 80]), activated keratinocytes expressing proinflammatory S100-proteins—MRP8 and MRP14 are seen in the rash of sJIA [29]. In another study by Frosch et al., MRP8 and MRP14 were found to be generalized and not limited only to the site of the rash; if the patient responded to treatment, MRP8 and MRP14 normalized [81]. In addition, during active disease, leukocytes were seen within the epithelium of sweat gland ducts [81].

Persistent fixed pruritic papules and plaques with fine scales were previously described and were reported to have unique histological findings of dyskeratosis in the superficial layers of the epidermis and minimal superficial dermal neutrophilic infiltrate [82]. Finally, malar rash in sJIA was described in a single case report [83].

### 6.4. Lymphadenopathy

Generalized lymphadenopathy is a common finding [8, 65]. In Behrens et al., 31% of the patients had initial presentation of lymphadenopathy [5]; in Modesto et al., 24% out of the good prognosis group and 51% of the bad prognosis group presented with lymphadenopathy [58]. The lymphadenopathy consists of painless rubbery mobile nodes and can be found in epitrochlear and axillary nodes [8, 65]; at times it can appear so striking that neoplasm may be suspected [84]. In comparison, mesenteric adenitis can be painful [8], and in the past it has led to operative interventions on children who were misdiagnosed with a surgical abdomen [65]. On radiographic studies, para-aortic adenopathy can be identified, and histologically, reactive changes are noted in the nodes [8].

### 6.5. Hepatosplenomegaly

Splenomegaly occurs in about 50% of the cases [8]; hepatomegaly does not occur as often [64], yet when it does, it frequently occurs when the disease is active [8]. Abnormal liver function can be seen prior to beginning therapy (but most patients did receive non-steroidal anti-inflammatory drugs for their ongoing fevers [85] which may cause this abnormality); however, clotting factors seem to be unaffected [8]. On histological examination periportal infiltrates of inflammatory cells were noted [85]. Hepatosplenomegaly needs to be monitored, as its progression may be related to amyloidosis [8].

### 6.6. Serositis

The most common type of serositis is pericarditis [8, 86]. In Behrens et al.'s study 10% of the patients presented with pericarditis [5]. According to Modesto et al., serositis (not specified) was seen in 14% of those who had good prognosis and 16% of those with bad prognosis [58]. It is typically recurrent but benign [87]. It often develops early in the course of sJIA and can manifest itself before the appearance of arthritis [8, 66, 87]. Children who have pericarditis may have nonspecific findings such as tachycardia and dyspnea, but may also have a friction rub [86]. Pericarditis may be an ominous sign of evolving myocarditits, which has more serious and potentially fatal complications of cardiomegaly, congestive heart failure, and arrhythmias [65, 88–90]. In a 1992 study by Goldenberg et al., which investigated symptomatic cardiac manifestations in JRA, 13 out of 172 patients were identified (11 of whom had sJIA); from the sJIA patients, pericarditis was recorded in 5, myopericarditis in 4 and isolated myocarditis in 2 patients [86]. Asymptomatic pleuritis and pleural effusions can present together with pericarditis or independently [8, 65]. 

Peritonitis is a rare manifestation of sJIA and was seen in two children, one during the first week of presentation, and the other 10 years after diagnosis [91].

### 6.7. Other Manifestations

Although rare, central nervous system manifestations such as seizures, meningismus, as well as irritability and decreased level of consciousness were previously described [92]. Ocular manifestations can be seen in sJIA, and uveitis is one of the complications [93]. A case report by Ishihara et al. described a patient with sJIA who developed bilateral panuveitis 3 years after her initial presentation [93]. In addition, Brown's syndrome (restricted movement of the superior oblique tendon) was seen in 3 children and reported in two case reports [94, 95]. Although nasal septum perforation is a complication of rheumatic illness, it was described in only 3 children with sJIA in a case series [96].

With the exception of pleuritis, pulmonary manifestations are also rare. Pulmonary function test abnormalities were reported in the 1980's by Wagener et al. [97]. In a cross-sectional study, Van Der Net et al. described restrictive pulmonary function in 8 out of 17 patients showing decreased total lung capacity; in 2 additional patients normal (yet lower) total lung capacity was seen [98]. Obstruction was not seen, as the Tiffeneau index (FEV1/FVC × 100%) was > 83% in all of the patients [98]. Interstitial pulmonary disease was reported by Athreya et al. [99]. Pulmonary hypertension was described in one case report [100], and pulmonary interstitial and intra-alveolar cholesterol granulomas were described in a 2001 case report [101].

The clinical presentation of sJIA and Kawasaki disease (KD) can be similar in young children. In a recent study from Binstadt et al., 5 out of 12 sJIA patients who had an echocardiogram that fully evaluated the coronary arteries on initial presentation had coronary artery dilation, and out of these, 2 patients had initially fulfilled the KD criteria [102]. Interestingly, a study by Maeno et al. showed that significant elevation of IL-18 levels was seen in sJIA, but not in KD or other types of JIA [103].

## 7. Differential Diagnosis

With nonspecific clinical and laboratory findings, the differential diagnosis of sJIA is extensive and should include infectious as well as post infectious etiologies, connective tissue diseases, vasculitis, malignancies, and autoinflammatory syndromes [42].

## 8. Complications

### 8.1. Amyloidosis

Serum amyloid A is an acute phase reactant which is elevated with inflammatory processes. It is the precursor for serum amyloid A protein [104, 105]. Amyloidosis is one of the most severe complications of sJIA. For unknown reasons, amyloidosis tends to be very rare in North America and yet affects a larger percentage of individuals in the UK and Turkey (7.4% and 16%, resp.) [106, 107]. Deposition of the protein has an effect on vital organs such as the kidney, liver, gastrointestinal tract, and heart [105]. Upon biopsy of rectal mucosa, subcutaneous fat, gum, or kidney, amyloidosis can be histologically recognized by using Congo red stain, which reveals eosinophilic deposition; when employing polarized light, the characteristic apple-green birefringence surfaces [105, 108, 109]. The first clinical sign of amyloidosis is proteinuria, but it is often missed and nephrotic syndrome is seen [8, 105]. Other symptoms that may suggest amyloidosis are: hypertension, hepatospenomegaly, and abdominal pain [8, 105]. Unless the inflammatory process of sJIA is successfully suppressed and amyloidosis reverses, death from progressive renal failure in those children with amyloidosis can result [8, 105]. Immonen et al. examined the long-term outcome of 24 patients with amyloidosis; sJIA was seen in 11/24 patients (46%). The overall 5 year survival rate was 88%, and 10-year survival rate was 75%. Out of the 24 patients with all subclasses of JIA, 10 died. Although the mortality for the different types of JIA was not specified, overall, a higher mortality was seen in patients who were treated solely with corticosteroids, while those who were treated with disease modifying antirheumatic drugs and/or cytotoxics had a better survival (*P* = 0.001) [110].

### 8.2. Macrophage Activation Syndrome

In 1985 Hadchouel et al. described a life-threatening complication of sJIA [111] for which the term MAS was later coined [112]. Uninhibited production and activation of both macrophages and T lymphocytes cause fever, rash, pancytopenia, hepatic insufficiency, coagulopathy, lymphadenopathy, and neurological dysfunction [113, 114]. MAS is not a unique entity, but rather a term used to describe a form of secondary HLH when it is seen in a rheumatic illness [115–117]. The incidence of MAS in the context of sJIA is estimated to be anywhere from 6.7%–13% [60, 118], and mortality rate ranges between 8–22% [118, 119]. As the symptomology of MAS is almost identical to that of sJIA, it is very difficult to diagnose. Some of the laboratory findings that are useful in distinguishing the two are the presence of cytopenias and normal ESR noted in MAS [118]. Nevertheless, it was recently shown that multiple sJIA patients had evidence of hemophagocytosis on bone marrow examination but did not have any clinical findings [60]. It is now believed that sJIA and MAS are possibly the two extremes of similar entities, where sJIA represents hidden or inactive MAS [4, 5].

## 9. Treatment

Historically, the management of sJIA included the use of nonsteroidal antiinflammatory drugs (NSAIDs), intravenous immune globulin (IVIG), corticosteroids, methotrexate, anti-TNF, cyclosporine, thalidomide, cyclophosphamide, and autologous stem cell transplantation [42, 120–123]. IVIG was initially encouraging, but in further studies, it was noted to perhaps be useful only for particular subsets of children with early systemic disease [124]. Although showing significant efficacy in other subtypes of JIA, methotrexate did not show adequate response in sJIA [125–127]. Anti-TNF agents were shown to have only a partial response [128–132]. With the recent expanding understanding of sJIA pathogenesis, the treatment has changed tremendously. A more targeted therapy in the form of biologic blocking agents transformed the treatment of sJIA [133, 134].

### 9.1. NSAIDs

In sJIA, NSAIDs are used for the management of pain, stiffness, and fever [135]. Historically, aspirin was used; however, the risk of intoxication [62] as well as development of Reyes syndrome promoted the replacement of aspirin with other NSAIDs [136–139]. Ibuprofen, meloxicam, naproxen, tolmetin, and celecoxib are approved by the Food and Drug Administration (FDA) for treatment of JIA [140]. Gastrointestinal adverse reactions such as gastritis and duodenitis are common [141, 142]. Pseudoporphyria associated with naproxen therapy can be seen in those children with light complexion and light hair. As permanent scarring may occur, awareness of this adverse reaction is important [143–145].

### 9.2. Corticosteroids and Cyclophosphamide

Although not considered to be disease modifying, systemic corticosteroids are often used when patients experience a preponderance of systemic features [135]. Kimura et al. studied the efficacy and side effects of high-dose alternate day prednisone and concluded that it was a valuable therapy with minimal adverse reactions [146]. 

Intravenous pulsed methylprednisolone is also useful in treating sJIA patients. In Adebajo and Hall's study, pulse steroids (30 mg/kg with a maximum of 1 g) was given to sJIA patients: 55% of the patients had full resolution of systemic manifestations, and 45% of the patients had reduction in arthritis; furthermore, 16% of the patients obtained remission [147]. Prolonged use of corticosteroids in children has multiple significant adverse reactions such as inhibition of growth, immunosuppression, striae, delayed puberty, osteoporosis, cushingoid habitus, myopathy, cataracts, hypertension, psychologic effects, and others, all of which can immensely affect the pediatric population [148–151]. Because of the considerable undesirable effects of corticosteroids, switching to an effective steroid sparing agent is critical in these patients. 

Humoral immunity is affected by high-dose cyclophosphamide [152], and high-dose corticosteroids cause decrease in E-selectin, ICAM-1 [153], CD11b, and CD18 in the synovial membrane and neutrophils [154]. Shaikov et al. described an open-label trial in 18 children with sJIA using combination methylprednisolone and cyclophosphamide, with significant improvement in systemic and articular manifestations [122]. Wallace and Sherry reported 4 children who improved after receiving intravenous pulse cyclophosphamide and methylprednisolone [121]. In 3 of the 4 patients, remission was obtained, and prednisone dose was decreased by ≥25%, and in all patients improvement was seen clinically (with ≥50% improvement in joint count and improved linear growth), as well as in their laboratory parameters [121]. Lehman reported 6 children treated with intravenous cyclophosphamide with minimal improvement [155]. Lastly, Chen et al. reported of 4 sJIA patients treated with intravenous cyclophosphamide and methylprednisolone; 2 of the patients achieved remission, 1 had shown improvement, and 1 did not improve [156].

### 9.3. Biologics

#### 9.3.1. IL-1 Inhibitors: Anakinra, Rilonacept, and Canakinumab

IL-1 inhibition can be achieved via 3 ways: IL-1 receptor antagonist, anakinra; IL-1R-IL1RacP-Fc fusion protein, rilonacept; or IL-1*β* antibody, canakinumab [157]. Initial reports using anakinra were promising with rapid improvement and remission of patients [133, 134]. However, later reports indicated that some patients did not respond as well to this treatment [158, 159]. In Gattorno et al.'s 2008 study, 10/22 (45%) of patients responded well to the therapy, 11/22 (50%) had incomplete response or no response to the therapy, and 1/22 (5%) could not be classified as either [159]. In Lequerré et al., at the last follow up, complete response was seen in 4/20 (20%), partial response seen in 5/20 (25%), and no response seen in 8/20 (40%) of patients (of the 3 patients not accounted for, 1 had a complete response at 3 months but did not have a reported follow up, and two were seen at two months with no response and did not have a reported follow up) [158]. In Ohlsson et al.'s study, 6/7 (86%) responded well to anakinra, while 1/7 (14%) did not have a good response [160]. In Zeft et al.'s 2009 study, 8/33 (24%) of patients did not have a good response [161]. A recent multicenter report of 46 patients treated with anakinra showed significant improvement; by 1 month of treatment, 86% of patients experienced abatement of fever and rash, and 84%, 63%, 83% and 71% of patients had normalization of CRP, ESR, ferritin levels, and platelet count respectively [162]. In that study complete response occurred only in 59% of the patients, partial response in 39% of the patients, and in 2% lack of response [162]. Two theories have risen to explain the different therapeutic response. Gattorno et al. postulated the existence of further classes in sJIA [159], and Nigrovic et al. hypothesized less efficacious blockade of IL-1 in an established disease secondary to either chronic inflammation (derived from ample supply of IL-1), or secondary to independent action of IL-17, possibly causing arthritis [162].

Rilonacept, the IL-1R-IL1RacP-Fc fusion protein (also known as IL-1 trap), showed immense response in an open label pretrial [163]. The selective IL-1*β* antibody canakinumab treats genetic fever syndromes, thus identifying this agent as a potential therapeutic modality for sJIA [164].

#### 9.3.2. Tocilizumab

Tocilizumab is a humanized monoclonal antibody, targeting both membrane bound and soluble IL-6 receptors [165]. By binding to these receptors, signal transduction through glycoprotein 130 is inhibited [166]. In 2003, Yokota reported the first encouraging use of IL-6 inhibition in children [166]. In 2005, a phase II trial with tocilizumab showed JIA 30%, 50%, and 70% improvement according to a core set of response variables in 10/11 (90.9%), 10/11 (90.9%), and 7/11 (63.6%) patients, respectively [167]. This definition of improvement was based upon the ACR Pediatric (ACR Pedi) 30 criteria, alternatively known as JRA, JIA, or Giannini's criteria of improvement [130, 168, 169]. It is an outcome measure for improvement defined as the following: a 30% improvement of at least 3 out of the following 6 core variables and no more than 30% worsening in one of them: (1) physician global assessment of disease activity; (2) parent/patient global assessment of overall well-being (each scored on a 10 cm visual analog scale); (3) functional ability; (4) number of joints with active arthritis; (5) number of joints with limited range of motion, (6) ESR [170]. Similarly additional outcome measures for improvement were later extrapolated: ACR pedi 50, 70, and 90, using the same guidelines as for the ACR Pedi 30 but defining a 50%, 70%, and 90% improvement in 3 of the 6 variables respectively, with no more than a 30% worsening in one variable [168, 169]. 

In 2005, an open-label phase II trial examining single ascending doses of tocilizumab had also shown good response: JIA 30%, 50%, and 70% improvement was seen in 11/18 (61%), 8/18 (44%), and 3/18 (17%) [171]. A 2008 study by Yokota et al. showed an ACR Pedi 30, 50, and 70 response rate in 51/56 (91%), 48/56 (86%), and 38/56 (68%), respectively at the completion of the open-label phase, where all patients received 3 doses of 8 mg/kg of tocilizumab every two weeks [172]. Out of the 56 patients, only 43 continued to the double-blind phase (3 patients developed antitocilizumab IgE antibodies, one had an anaphylactoid reaction, one had a gastrointestinal hemorrhage, and one had lack of efficacy). It was reported that in comparing the tocilizumab treatment group and the placebo group, the ACR Pedi 30, 50, and 70 responses were: 16/20 (80%), 16/20 (80%), 15/20 (75%) and 4/23 (17%), 4/23 (17%), and 3/23 (13%), respectively. In the open-label extension of the trial, ACR Pedi 30, 50, and 70 were achieved by 47/48 (98%), 45/48 (94%), and 43/48 (90%), respectively [172]. Lastly, in a recent phase 3 trial, comparing tocilizumab treatment group and placebo group after 12 weeks of therapy, De Benedetti et al. reported absence of fever and JIA ACR 30 to be 85% versus 24% (*P* < 0.0001); furthermore, JIA ACR 50, 70, and 90 were compared between the treatment and placebo groups and were 64/75 (85%), 53/75 (71%), 28/75 (37%) versus 11/37 (11%), 3/37 (8%), 5/37 (2%), respectively [173]. In April 2011, the FDA approved the use of tocilizumab in sJIA patients older than 2 years of age [174]. 

#### 9.3.3. Abatacept

Abatacept is a fusion protein that blocks the CD80 or CD86 interaction with CD28, which alters the costimulatory signal, thus inhibiting T-cell activation [175]. In 2008, a study by Ruperto et al. showed ACR Pedi 30% or more improvement in 65% of the systemic arthritis group, but the study excluded children with active systemic manifestations for the preceding 6 months [168]. In Ruperto et al.'s long-term extension, abatacept was again reported to have good response rate; the ACR Pedi 30, 50, 70, 90 and inactive disease response rate in patients with sJIA without systemic manifestations were 88%, 88%, 63%, 13%, and 25% correspondingly [176]. In a later report that year, Ruperto et al. discerned improvement in health-related quality of life (HRQOL) in JIA patients treated with abatacept (in which about 20% of the patients that were studied had sJIA) [177].

#### 9.3.4. Combination Therapy Anakinra and Abatacept

An anecdotal report of a combination therapy of anakinra and abatacept in 4 children with recalcitrant sJIA described improvement of their symptoms, with no significant adverse reactions [178].

#### 9.3.5. Antitumor Necrosis Factors Antibodies (Anti-TNF)

There are three different types of antitumor necrosis factor (anti-TNF) therapies: etanercept: a soluble TNF*α* receptor [169, 179, 180], Infliximab: a chimeric monoclonal TNF*α* antibody, and Adalimumab: a humanized monoclonal antibody.

The results from Lovell et al.'s 2000 study comparing sJIA patients' flare rate between placebo and etanercept therapy were encouraging, with 7/8 (88%) patients on placebo having a flare, and 4/9 (44%) of those on etanercept having a flare (statistically significant *P* < 0.001) [181]. Several later studies show that patients with sJIA appear to have only partial response to anti-TNF agents [128–132]. 

In 2003 Lovell et al. published interim results from an ongoing multicenter study examining etanercept, and reported improvement rates in JRA (30%, 50% and 70%). In the per protocol group at the end of the 2nd year, 30% improvement was seen in 10/12 patients (83%), 50% improvement was seen in 9/12 patients (75%), and 70% improvement was seen in 8/12 patients (67%); in their modified intent-to-treat group (which included the patients who discontinued therapy) 30% improvement was seen in 10/17 patients (59%), 50% improvement was seen in 9/17 patients (53%), and 70% improvement was seen in 8/17 patients (47%) [169]. In Horneff et al.'s 2004 study, a lower efficacy was seen in sJIA patients, where Giannini's criteria of 30%, 50%, and 70% improvement was seen in 48%, 33%, and 11% of the patients, respectively, after 1 month of treatment with etanercept, and after 3 months of treatment, improvement was seen in 63%, 39%, and 24% [130]. In Kimura et al.'s 2005 study examining etanercept therapy, 37/82 (45%) patients had poor response (<30% improvement), 7/82 (9%) had a fair response (30–<50% improvement), 11/82 (13%) had a good response (50–<70% improvement), and 27/82 (33%) had an excellent response (>70% improvement), where the response was defined as a percentage decrease from baseline of the following: steroid dose, count of actively involved joints, inflammatory markers (ESR, CRP, or platelet count), and physician global assessment of disease activity score, rather than the ACR pedi response criteria [131]. In Russo and Katsicas' 2009 study, patients were treated initially with etanercept, but if improvement was not seen, patients were switched to therapy with either infliximab or adalimumab. The ACR pedi 30, 50, 70, and 90 criteria were used to assess clinical improvement and were seen in 35 (78%), 28 (62%), 21 (47%), and 14 (31%) of patients, respectively [132]. See Table 2. In Quartier et al.'s 2003 study, it was recognized that in comparing the 30% improvement rate between sJIA and oligoarticular or polyarticular JIA, those with sJIA had a greater likelihood of not reaching the 30% improvement (with *P* values of 0.0002 and 0.0031, resp.). When comparing the 50 and 70% improvement rate, sJIA also had a significant risk of not attaining that improvement level in comparison with oligoarticular onset JIA but did not differ in risk value from polyarticular onset JIA [129].

In a 2003 Lovell et al.'s study it was reported that out of the 5 sJIA patients who withdrew from the study, 4 had suboptimal clinical response, and 1 had an adverse event [169]. In the 2006 extension study, it was reported that 19 patients entered the extension study, and only 6 patients stayed in the extension study for ≥4 years (3 of the 13 patients withdrew secondary to lack of efficacy) [179]. In the 2008 open-label extension it was reported that 19 patients had entered the trial, but only 5 entered the 8th year [128], In Horneff et al.'s 2004 study, 17 of the 66 sJIA patients enrolled withdrew from the trial, where inefficacy of therapy was the reason for discontinuation in 14 out of the 17 patients, adverse effects were seen in 2 patients and 1 patient withdrew for other reasons [130]. In Kimura et al.'s 2005 study, disease flares were seen in 37/82 patients (45%) in all levels of therapeutic response; however, they were more likely to occur in those who responded poorly to treatment (25/37 patients (68%)) than in those who had an excellent response (7/27 patients (26%)). Cessation of treatment occurred in 29/82 patients (35%) mainly secondary to inefficacy or flare in 72.4% of these patients [131]. Tynjälä et al.'s 2009 study looked at length of anti-TNF therapy usage (either etanercept or infliximab). At 24 and 48 months 46% and 76% of patients, respectively, had discontinued their medications. Inefficacy was the most common reason for discontinuation in the sJIA group [182]. 

In Katsicas and Russo's 2005 study, patients who had previously failed therapy with etanercept were treated with infliximab. It was reported that the majority of patients did not reach improvement with infliximab; however, the one patient who showed a response to infliximab did not have systemic manifestations at the onset of therapy [183]. A statistical significance (*P* = 0.03) was recorded in Russo and Katsicas' 2009 study between remission and both the absence of systemic manifestations at the onset of anti-TNF therapy and improvement after 3 months of therapy. In that study 64% (29/45) of patients showed an improvement after 3 months of treatment, and 73% (33/45) of patients displayed an improvement after 6 months [132].

#### 9.3.6. Rituximab

Rituximab is a chimeric monoclonal antibody against CD20, targeting B cells. Wouters et al. described a higher B-cell activity in all types of JIA, including sJIA [184]. There are several case reports detailing treatment of sJIA with rituximab. Kasher-Meron et al. describe an 18-year-old female with a 12-year history of sJIA that was recalcitrant to therapy that responded well to therapy with rituximab [185]. A case series by Narváez et al. described three adult patients with unrelenting sJIA (duration of disease between 18–27 years), all of whom had noteworthy improvement with rituximab therapy; with the exception of one patient with hypersensitivity reaction, no other significant adverse reactions were seen [186]. Lastly Feito and Pereda described an 8-year-old female that responded well to rituximab, in both systemic manifestations and articular manifestations [187].

### 9.4. Cyclosporine

Cyclosporine is an immunomodulator that inhibits the synthesis of IL-1, IL-2, TNF-*γ*, and *α*-interferon [188–190]. The results of a 10-year prospective study looking at the efficacy of cyclosporine A showed benefit for some children with sJIA, but for the majority complete remission was not achieved [191]. In a later surveillance study, out of those patients who were still receiving cyclosporine at their last reported visit, only 5% have achieved full clinical response, while 63% had mild to moderate activity and 32% had severe uncontrolled disease [192]. Associated adverse reactions reported are hypertrichosis, elevated serum creatinine levels, gingival hyperplasia, gastrointestinal irritation, and hypertension [192].

### 9.5. Thalidomide

Thalidomide prevents cytokine synthesis by disturbing mRNA synthesis rather than blockade [193], and is a known anti-inflammatory agent that suppresses angiogenesis, cell adhesion molecule expression, TNF-*α*, IL-1, IL-6, and nuclear factor-*κ*G [194–196]. In 2002, Lehman et al. reported on 2 children with intractable sJIA who were treated with thalidomide therapy and had significant improvement [197]. In 2004, Lehman et al. reported of 13 additional children who were treated with thalidomide. A response was seen in 11 children, and 10 of them had JRA improvement scores ≥50% in concordance with the preliminary definition of improvement in juvenile arthritis [120, 170]. Statistically significant decrease in prednisone dosage, decrease in ESR, and increase in hemoglobin level were seen [120]. In 2007 a 3-patient case series was reported by Garc*ί*a-Carrasco et al., where after therapy with thalidomide, 3 recalcitrant patients entered remission [198].

## 10. 2011 American College of Rheumatology Recommendations

In the recent 2011 American College of Rheumatology recommendations for the treatment of sJIA, the recommendations were made by identifying the patient as belonging to one out of two distinct clinical groups: active systemic features (without active arthritis), or active arthritis (without active systemic features), and also by disease activity level and by prognosis. For those patients with both active systemic features and active arthritis, a recommendation was not made, but use of the two recommendations was suggested. Furthermore, recent therapeutic agents, such as IL-6 inhibitors and other IL-1 inhibitors besides anakinra were not included the recommendations as they were not available [199].

For systemic arthritis with active systemic features but no arthritis, initiating NSAIDs, systemic glucocorticoids, or anakinra as initial therapy is dependent on disease activity and prognostic features. Patients with low disease activity and good prognostic features are recommended the treatment of NSAIDs, followed by glucocorticoids and anakinra. NSAIDs may be omitted for those patients with either poor prognostic features or high disease activity. For patients with high disease activity and without poor prognostic features, initial therapy with systemic glucocorticoids followed by anakinra when not responding well is recommended. For patients with poor prognostic features, initial therapy may be either systemic glucocorticoids or anakinra [199]. Methotrexate was deemed inappropriate for this group, and both thalidomide and calcineurin inhibitors were of uncertain benefit [199]. 

Treatment recommendations for systemic arthritis with active arthritis but without active systemic features include up to 1 month of NSAIDs with glucocorticoid joint injections. If no improvement or worsening, methotrexate was the next therapy. After 3 months of methotrexate therapy, dependent on disease activity, the patient can start on either TNF*α* inhibitor or anakinra. After 4 months of TNF*α* therapy, if the disease activity is still high or moderate (but with poor prognosis), abatacept was recommended. Calcineurin inhibitors were found to be unsuitable for this group of patients [199]. 

## 11. Course, Prognosis, and Outcome

The course and outcome of sJIA can vary considerably, ranging from a monocyclic course with good outcome, to a more complicated one which involves considerable morbidity or mortality. In approximately half the patients with sJIA, a monocyclic course is seen, and complete recovery with minimal physical limitations can be achieved within 2–4 years [42, 69, 200]. Waning flares of systemic involvement and mild arthritis can be seen in those with relapsing course [42]. Some patients achieve resolution of their systemic features, but suffer from significant persistent arthritis which tends to resolve after about 5 years [201]. However, approximately 30% of the patients suffer from devastating destructive chronic polyarthritis that is responsible for most of the morbidity and account for the worst prognosis in this disease [69, 202]; resolution of the arthritis does not usually occur by adulthood [42]. These patients tend to have more severe systemic manifestations [203], and for about 23–30%, systemic features persist for more than 10 years after initial presentation [204, 205]. 

Systemic manifestations 6 months following presentation, thrombocytosis [206], hip involvement in the setting of polyarthritis, and generalized lymphadenopathy in those less than 8 years of age [58] are predictors of poor outcome. Several studies have attempted to stratify the risk for development of destructive arthritis suggest that early course arthritis of the hips, cervical spine, and small joints of the digits can indicate higher risk [203, 207]. In the past, amyloidosis was a significant risk factor for death [42], but according to Immonen et al., new onset of amyloidosis in sJIA was not seen in Finland since 1991 [110]. MAS is a significant complication, and mortality was seen in 8–22% [118, 119]. Lastly, psychological complications such as depression, anxiety, and social isolation are important patient outcomes [208].

## 12. Conclusion

The clinical symptoms of sJIA have not changed significantly from 1897 when it was first described by Still. The recognition of the unique nature of sJIA in comparison to other types of JIA as well as an increased collective understanding of the pathogenesis, instigated significant advancement in treatment options offered to these children. IL-1 blockade revolutionized the treatment and outcome for sJIA patients. With the discovery of novel and targeted biologics, the pediatric rheumatologist is presented with several choices. In a patient who presents initially with arthritis and systemic features, NSAIDS, steroids, and IL-1 blockers are reasonable considerations depending upon the severity of symptoms and the need for a prompt remission of symptoms. After the initial presentation, for those patients with primarily systemic features, we would continue IL-1 blocker and taper steroids as tolerated. For milder cases with systemic features, thalidomide remains an option. Tocilizumab which was recently approved for sJIA is a choice for those patients that have more severe disease or fail to respond to IL-1 blocking agents, but it remains an indefinite commitment to every two-week infusions at present. For children with a predominantly polyarticular course who no longer have fever and rash, we would consider anti-TNF agents and methotrexate. An individualized approach for each patient is recommended.

The newly gained knowledge and the development of new treatments are changing the lives of children who are suffering from sJIA today. Their prognosis and disease outcome are much better than in previous generations. There is still ample knowledge to be learned in order to create better and more effective therapies.

## Figures and Tables

**Table 1:** Incidence of sJIA (per 100,000/year) in the literature.

Study	sJIA incidence 100,000/year
Modesto et al. 2010 [7]	0.5
Pruunsild et al. 2007 [10]	0.9
Berntson et al. 2003 [11]	0.6
Huemer et al. 2001 [12]	0.4
Kaipiainen-Sepp*ӓ*nen and Savolainen 2001 [13]	0.9
Malleson et al. 1995 [14]	0.49
Pelkonen et al. 1994 [15]	0.47

**Table 2:** Response to anti-TNF therapy in sJIA patients.

Study	Response	Number of patients	Discontinuation
Lovell et al. 2003 [169] (per protocol group*) Etanercept	At 24 months: (i) JRA 30% definition of improvement seen in 83% of patients. (ii) JRA 50% definition of improvement seen in 75% of patients. (iii) JRA 70% definition of improvement seen in 67% of patients.	12	Not included

Lovell et al. 2003 [169] (modified intent-to-treat group**) Etanercept	At 24 months: (i) JRA 30% definition of improvement seen in 59% of patients. (ii) JRA 50% definition of improvement seen in 53% of patients. (iii) JRA 70% definition of improvement seen in 47% of patients.	17	5 (29%)

Horneff et al. 2004 [130] Etanercept	At 1 month: (i) Giannini's criteria of 30% improvement seen in 48% of patients. (ii) Giannini's criteria of 50% improvement seen in 33% of patients. (iii) Giannini's criteria of 70% improvement seen in 11% of patients. At 3 months: (i) Giannini's criteria of 30% improvement seen in 63% of patients. (ii) Giannini's criteria of 50% improvement seen in 39% of patients. (iii) Giannini's criteria of 70% improvement seen in 24% of patients.	66	17 (26%)

Kimura et al. 2005 [131] Etanercept	Mean duration of treatment: 24.8 ± 12.3 months (3–70 months): (i) Poor response (<30%) seen in 45% of patients. (ii) Fair response (30 to <50%) in 9% of patients. (iii) Good response (50 to <70%) seen in 13% of patients. (iv) Excellent response (>70%) seen in 33% of patients.	82	29 (35%)

Russo and Katsicas 2009 [132] Etanercept initially, if no improvement seen, infliximab or adalimumab were studied.	Treatment for at least 6 months: (i) ACR Pedi 30 seen in 78% of patients. (ii) ACR Pedi 50 seen in 62% of patients. (iii) ACR Pedi 70 seen in 47% of patients. (iv) ACR Pedi 90 seen in 31% of patients.	45	22 (49%) of patients switched to a second anti-TNF (either infliximab-17 patients, or adalimumab-5 patients) secondary to lack of response in 9 pts and lack of efficacy subsequently. Infliximab was then discontinued in 4 patients secondary to lack of efficacy and in 6 patients secondary to toxicity.

*Per protocol group consisted of 43 patients (with pauciarticular, polyarticular or systemic JRA) who were treated with etanercept for 2 years at time of Lovell et al.'s analysis. Out of the 43 patients in this group, 12 patients had systemic JRA.

^∗ ∗^Modified intent-to-treat group consisted of 51 patients (with pauciarticular, polyarticular or systemic JRA): 43 were included in the per protocol group, 7 withdrew secondary to an inadequate clinical response (of these, 4 had systemic JRA), and 1 withdrew secondary to an adverse reaction (that patient had systemic JRA). Out of the 51 patients in this group, 17 patients had systemic JRA.
